# Peroxisomal Malfunction Caused by Mitochondrial Toxin 3-NP: Protective Role of Oxytocin

**Published:** 2019

**Authors:** Mehdi Moslemi, Fereshteh Motamedi, Sareh Asadi, Fariba Khodagholi

**Affiliations:** *Neuroscience Research Center, Shahid Beheshti University of Medical Sciences, Tehran, Iran.*

**Keywords:** Oxytocin, Huntington disease, 3-NP, Peroxisome, Oxidative stress, Pex14, PMP70

## Abstract

Peroxisomes are single membrane cell organelles with a diversity of metabolic functions. Here we studied the peroxisomal dysfunction and oxidative stress after 3-nitropropionic acid (3-NP) induced neurotoxicity and the possible protective effects of oxytocin. Adult male and female rats were subjected to OXT and/or 3-NP treatment. The antioxidant enzymes, Superoxide dismutase (SOD) and Catalase (CAT) activities as well as expression level of Peroxin 14 (Pex14), a marker for peroxisomal number and Peroxisomal membrane protein of 70 kDa (PMP70), a metabolic transporter in peroxisome in different brain regions of both sexes were studied. The results indicated that 3-NP significantly decreased the expression level of Pex14 and PMP70 in various studied areas in male and female rats. In addition, 3-NP reduced the SOD and CAT activity in different brain regions in both sexes. OXT treatment increased the expression level of peroxisomal proteins Pex14 and PMP70 which are representative of peroxisome performance improvement. Besides, it ameliorated the antioxidant system capability through increasing the activity of the SOD and CAT in all studied brain regions including Striatum, Hippocampus, Prefrontal Cortex and Amygdala with no differences in male and female rats. This study demonstrated that toxin 3-NP, could ultimately cause peroxisomal malfunction and so determines the contribution of peroxisomal dysfunction in the etiology of HD pathology. OXT significantly increased peroxisomal function and antioxidant system defense capability, therefore illustrates that OXT might be an alternate treatment approach for the neurodegenerative diseases like HD.

## Introduction

Peroxisomes are substantial organelles found in most eukaryotic cells which contain H_2_O_2_-producing oxidase and the H_2_O_2_-detoxifying enzyme catalase (1). Peroxisomes have an important role in metabolic and anabolic functions, including fatty acid oxidation and lipid biosynthesis through different enzymatic activities. Comprehensive functions of peroxisomes are the oxidative metabolism of fatty acids and the degradation of H_2_O_2 _by catalases (2).

Mitochondria and peroxisomes are metabolically linked organelles since they share common pathways such as fatty acid β-oxidation, glyoxylate detoxification or special fatty acids degradation via α-oxidation (3-5). It has been recently shown that both organelles have an important role in the production and elimination of ROS and there is a close crosstalk between these organelles in the intracellular ROS homeostasis (6, 7). However, other ROS detoxifying enzymes including superoxide dismutases, peroxiredoxins, glutathione S-transferases, and epoxide hydrolases are known in higher eukaryotes to contribute to peroxisomal redox balance (6). Also, it is suggested that extensive peroxisomal *α*-oxidation depends on the ATP supply from the mitochondrial respiratory chain. 

3-Nitropropionic acid (3-NP), a natural toxin, has been reported to induce neuronal degeneration in the striatum (ST), cerebral cortex, and hippocampus (HIP) (8, 9). 3-NP-induced neurotoxicity is due to irreversible inhibition of succinate dehydrogenase, thereby blocking both the Krebs cycle and complex II of the mitochondrial electron transport (10). It was suggested that mechanisms of 3-NP toxicity have often been associated with glutamate-mediated excitotoxicity (11), blockade of ATP and abnormal Ca^2+^ influx (12), caspase-3 and many evidences suggest that neurotransmission systems play a key role in the 3-NP toxicity (13, 14). The impairment of energy production results in increased level of free radicals and predisposes neurons to excitotoxic damage (15). The 3-NP-induced neurotoxicity model is also a reliable tool for studying HD because some of the effects involved in striatal degeneration triggered by 3-NP could replicate some of the mechanisms of cell death involved in HD patients and the introduced histological lesions and behavioral changes are similar to those manifested in HD patients. In 3-NP induced neurotoxicity, increased oxidative damage has been consistently observed (16). The ROS accumulation in neurons, and subsequent oxidative stress were moderated by enzymatic or non-enzymatic antioxidants. Enzymatic antioxidants including superoxide dismutase (SOD), glutathione peroxidase (Gpx), and catalase (CAT) are defense mediator against free radicals. Non-enzymatic antioxidants are represented by ascorbic acid (Vitamin C), α- tocopherol (Vitamin E), and glutathione (GSH). Previous studies demonstrated that therapeutic antioxidant approaches like CoQ10, in the 3-NP induced mitochondrial toxicity, resulted in dose-dependent neuroprotection, with significant reductions in striatal lesion (17). So it is assumed that the 3-NP induced mitochondrial impairment increased peroxisomal dysfunction and consequently increased the oxidative stress and apoptotic damage in the neurodegeneration, as described previously (18, 19). OXT, a neurohypophysial nonapeptide, exerts various central and peripheral effects through stimulation of different signaling pathways including induction of uterine contractions during parturition and milk ejection and specific behavioral effects (20-22). Previous studies demonstrated that the metabolic properties of OXT and suggested that central OXT promotes anabolic metabolism and growth through regulating of insulin levels (23, 24). In addition, antioxidant properties of OXT have been established by other groups (25-27). Araceli *et al.* tested that OXT treatment reduces the lethal reperfusion injury of cardiomyoblasts and inhibits ROS production in cells exposed to hypoxia (26). Furthermore, behavioral and neuronal effects of OXT are often sex-specific. For example, intracerebroventricular administration of OXT reversed social defeat-induced social avoidance in male (28), but not in female rats (29). Therefore, this investigation was designed to answer the possible involvement of peroxisome in HD onset. For this purpose 3-NP as a mitochondrial toxin was used. So the functional connection of mitochondria and peroxisome could be evaluated in the context of HD-like symptoms as well. In the next step the effect of OXT as a treatment on this connection was evaluated. For this aim the amounts of peroxisome related proteins were assessed through western blot and enzymatic methods considering sex differences in various brain regions including ST, HIP, Amygdala (AMY), and prefrontal cortex (PFC).

## Experimental

*Animals*

Virgin male and female Sprague–Dawley rats, three months old weighting 220-250 g, were obtained from Pasteur Institute (Tehran, Iran). All experimental procedures were approved by the Ethics Committee of Shahid Beheshti University of Medical Sciences. The study received written ratification from the Neuroscience Research Center Ethics Board (IR.SBMU.PHNS.REC.1397.016). All animals were treated in accordance with the guidelines outlined in the Care and Use of Laboratory Animals (NIH publication, 85-23, revised 1996). Four animals were housed in each cage with free access to chow and water. The rats were preserved on a standard 12 h light/12 h dark cycle, at a permanent temperature (25 ± 2 °C). 

*Experimental Design*

The animals were randomly divided into 4 male and 4 female experimental groups:

Control group (n = 6), animals received isotonic saline solution as a vehicle.

3-NP group (n = 6), administrated with 3-NP (20 mg/kg/day) via intraperitoneal (i.p).

OXT group (n = 6), OXT was injected (10 ng/µL) intracerebroventricular (ICV) based on our pilot studies. 

3-NP+OXT group (n = 6), OXT injected 24 h before 3-NP administration. 

This grouping was performed in both sexes. 3-NP (20 mg/kg/day) administrated via intraperitoneal for 5 days and 7 day, in male and female rats respectively. The rationale for 3-NP dosage and administration was the methods described previously (30-32).

Then animals decapitated and brain areas including ST, AMY, HIP, and PFC were rapidly separated. Tissues were immediately frozen in liquid nitrogen, and stored at −80 °C for subsequent enzymatic and molecular analyses.

*Western blot assay*

The frozen brain tissues (ST, AMY, HIP, and PFC) were homogenized in lysis buffer augmented with a complete protease at 4 °C, and protein concentrations were determined using the colorimetric method of Bradford (33). The samples (60 µg per gel lane) were separated by electrophoresis on 12% sodium dodecyl sulfate (SDS)-polyacrylamide gels and electro-transferred to polyvinylidene difluoride (PVDF) membranes. The used antibodies for immunoblotting are PMP70 antibody (abcam, 1:1000), Pex14 antibody (abbexa. 1:1000) and secondary HRP conjugated anti-rabbit (Cell Signaling Technology, 1:3000). Then, Amersham ECL kit (GE health care) was used to identify immunoreactive polypeptides and eventually, the results were accounted by the scan of X-ray films and analysis by ImageJ software. β-actin (1:1,000; Cell Signaling Technology) was used as an internal control.

*Enzymatic assays*

*Catalase (CAT) activity assay*

CAT activity was determined according to the method described by Goth with some modification (34). Briefly, H_2_O_2_ (0.01 M) was added to 60 µg of homogenized tissue sample. The enzymatic reaction was stopped with ammonium molybdate and the break down rate of H_2_O_2_ as the activity of catalase was monitored at 405 nm, spectrophotometrically.

*SOD activity assay*

SOD activity was measured based on the method of Kakkar *et al.* (35). The homogenized tissue samples were added to assay mixture containing sodium pyrophosphate buffer (pH 8.3, 0.05 M), phenazine methosulphate, and nitroblue tetrazolium. Nicotinamide adenine dinucleotide (NADH) was added to start the reaction and then stopped by adding glacial acetic acid. Color intensity was measured spectrophotometrically at 560 nm.

*Statistical analysis*

Experimental data were analyzed using GraphPad Prism (V5; GraphPad Software) and the standard one-way analysis of variance (ANOVA) followed by multiple comparisons post hoc Tukey test was performed to compare the statistical significance between groups. *P*-value < 0.05 was considered statistically significant.

## Results

*OXT enhanced Pex14 level, which was decreased by 3-NP in different brain regions of male and female rats*

**Figure 1:**
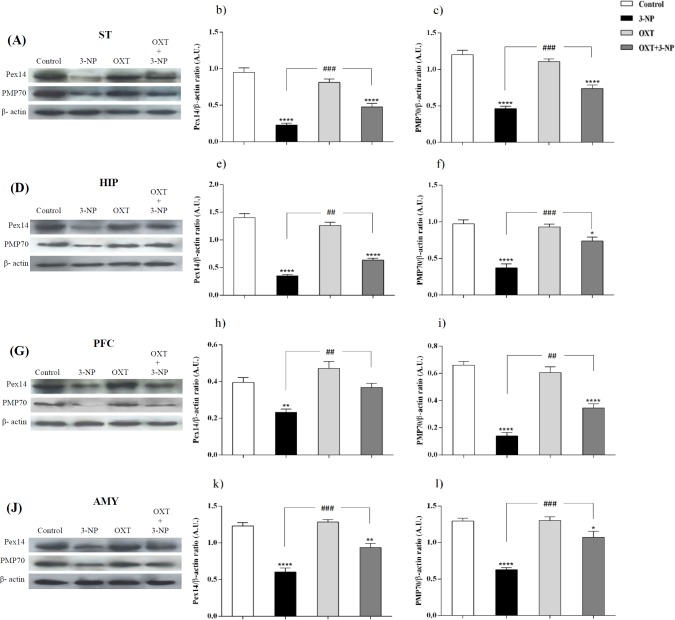
Effect of OXT on expression level of Pex14 and PMP70 in the different brain regions of 3-NP injected male rats. 3-NP reduced Pex14 (b, e, h and k) and PMP70 (c, f, i and l) protein levels in the studied brain regions in comparison with the Control group while OXT improved this effects in all studied brain areas of male rats. Data are presented as means ± SEM. (n = 6/group). ^*^p < 0.05, ^**^p < 0.01, ^***^p < 0.001, ^****^p < 0.0001 compared with the Control; ^##^*p *< 0.01, ^###^*p *< 0.001 compared between the 3-NP and 3-NP-OXT groups. OXT: oxytocin; 3-NP: 3-Nitropropionic acid; Pex14: peroxin 14; PMP70: peroxisomal membrane protein of 70 kDa

**Figure 2:**
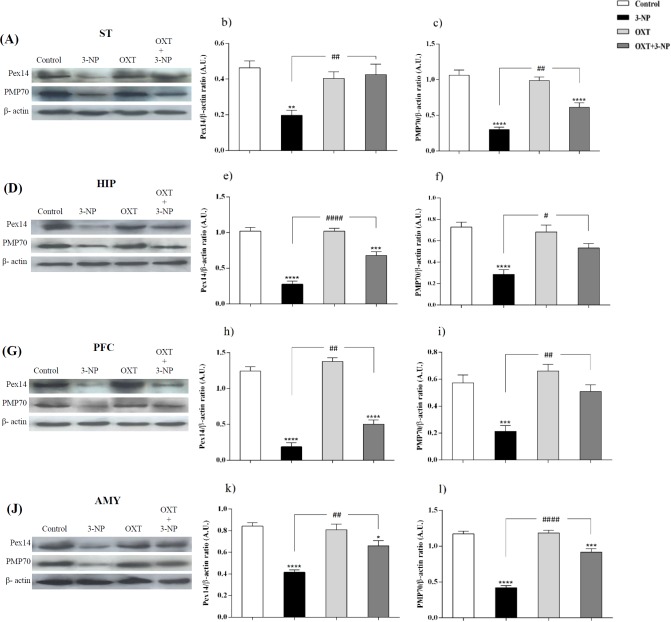
Effect of OXT on expression level of Pex14 and PMP70 in the different brain regions of 3-NP injected female rats. 3-NP reduced Pex14 (b, e, h and k) and PMP70 (c, f, i and l) protein levels in the studied brain regions of female rats in comparison with the Control group while OXT improved this effects in all studied brain areas of female rats. Data are presented as means ± SEM. (n = 6/ group). *p < 0.05, **p < 0.01, ***p < 0.001, ****p < 0.0001 compared with control; #*p *< 0.05, ##*p *< 0.01, ###*p *< 0.001 compared between the 3-NP and 3-NP-OXT groups. OXT: oxytocin; 3-NP: 3-Nitropropionic acid; Pex14: peroxin 14; PMP70: peroxisomal membrane protein of 70 kDa

Figures 1 and 2, showed that 3-NP significantly decreased Pex14 levels, as a peroxisomal number marker, in the ST, PFC, HIP, and AMY in male rats (36). In those rats that received 3-NP, OXT enhanced the amount of Pex14. The same results were obtained in different brain regions of male rats mentioned above. OXT administration had no effect when used alone (Figure 1). In female rats a similar pattern was observed so that, 3-NP administration decreased Pex14 level and OXT could increase it. This result was repeated in the ST, HIP, PFC and, AMY of females.

**Figure 3:**
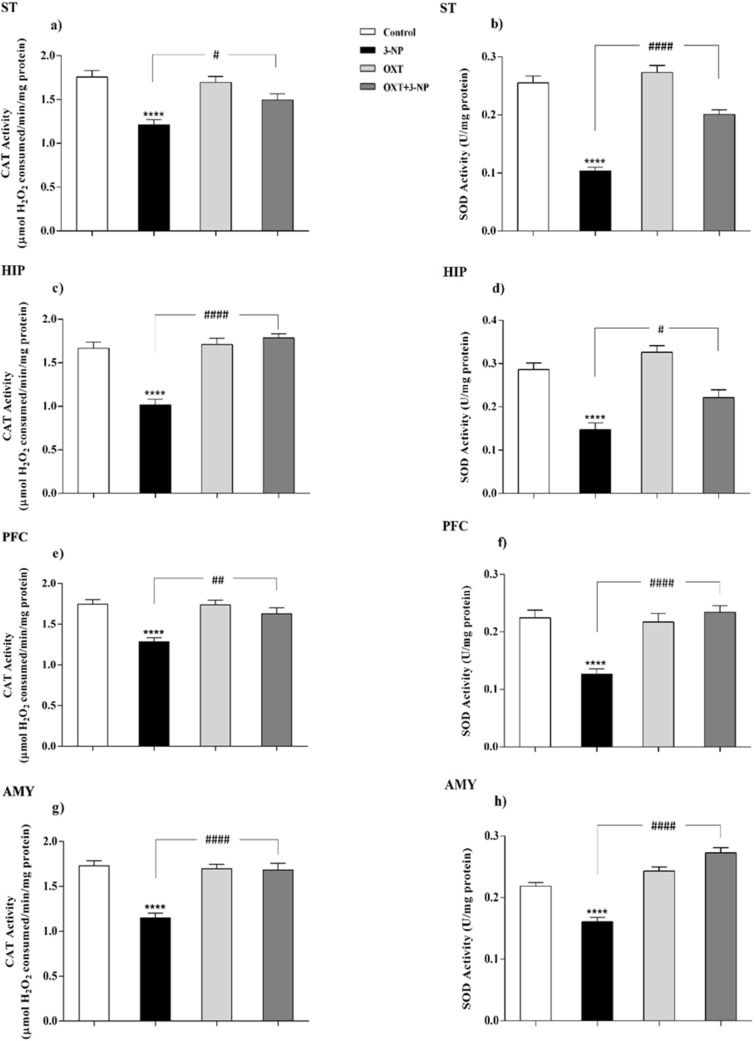
Effect of OXT administration on the activity of CAT and SOD enzymes in different brain regions of 3-NP injected male rats. 3-NP reduced the activity of the CAT in different brain regions but interesting, OXT improved its activity in all areas studied in male rats (a, c, e and g). Reduction of activity of the SOD, which was caused by toxin injection, was also improved by OXT in different areas of male rats (b, d, f and h). Data are presented as means ± SEM (n = 6/group). ****p < 0.0001 compared with the Control; #*p *< 0.05, ##*p *< 0.01 and ####*p *< 0.0001 compared between the 3-NP and 3-NP-OXT groups. OXT: oxytocin; 3-NP: 3-Nitropropionic acid; CAT: catalase; SOD: superoxide dismutase

**Figure 4:**
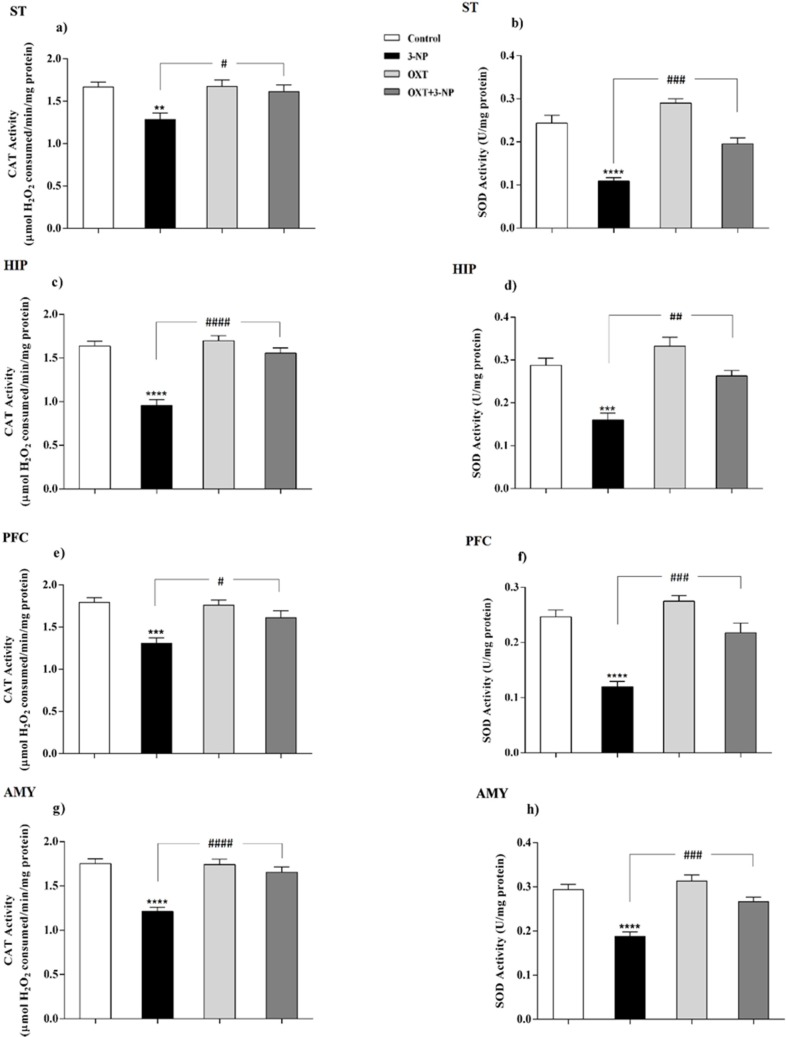
OXT effect on CAT and SOD activity in different brain areas of female rats that received 3-NP. (a, c, e and g) 3-NP significantly reduced CAT activity in the various brain regions of the female rats, but OXT improved the effect of 3-NP in all studied regions. (b, d, f and h) OXT stand against activity reduction of the SOD, which was followed by 3-NP injection, in different brain areas of female rats. Data are presented as means ± SEM (n = 6/group). **p < 0.01, ***p < 0.001, ****p < 0.0001 compared with the Control; #*p *< 0.05, ##*p *< 0.01 and ####*p *< 0.0001 compared between the 3-NP and 3-NP-OXT groups. OXT: oxytocin; 3-NP: 3-Nitropropionic acid; CAT: catalase; SOD: superoxide dismutase

*OXT raised PMP70 level, which was decreased by 3-NP in different brain regions of male and female rats*

Our results indicated (Figures 1 and 2) that 3-NP caused a decline in PMP70 levels in the different brain regions of the male rats. Simultaneously, OXT was found to be able to compensate this effect and significantly increased PMP70 levels as a metabolic transport of long and branched-chain fatty acyl-CoAs in all studied brain areas of male rats (37). But OXT injection had no effect when used alone in male rats. 

Similar to the pattern was seen in the male rats, in the female rats, 3-NP diminished PMP70 level in different studied brain regions. But OXT intensified PMP70 levels in studied brain areas of female rats that received 3-NP. OXT lonely had no effect on the PMP70 levels in all mentioned brain regions of female rats. 

*OXT increased the activity of CAT that decreased by 3-NP*

As shown in Figures 3 and 4, 3-NP injection significantly decreased the CAT activity in the ST, HIP, PFC, and AMY in male rats. In these conditions, OXT significantly enhanced the activity of CAT in those male rats that received 3-NP in all studied areas. But OXT had no effect when used alone. Our results demonstrated a similar pattern of OXT effects in the females and 3-NP dropped the CAT activity in different studied brain regions that mentioned above. OXT elevated the CAT activity in those female rats that received 3-NP. However, OXT injection lonely had no effect in the CAT activity in studied brain regions of females. So, our results indicated that OXT could stand against 3-NP induced decreased CAT activity both in male and female rats in all studied brain areas.

*OXT intensified the activity of SOD attenuated by 3-NP*

Our results showed (Figures 3 and 4) 3-NP has significantly reduced the activity of SOD in the studied brain regions that were mentioned above. OXT Injection prevented the effects of 3-NP and increased the activity of the SOD.But, when OXT was used alone, there was no significant effect on the activity of SOD in the brain areas of the male rats. The same effect was observed in all studied areas of female rats. 3-NP decreased the activity of SOD in all studied brain regions. But OXT in all studied regions caused a protective effect in 3-NP induced SOD diminished activity and increased its activity. So, our results showed the protective effect of OXT on the decreased SOD activity induced by the mitochondrial toxicity of 3-NP in the animal model of HD-like disease. 

## Discussion

Peroxisomes are essential in cellular metabolism and their deficiency causes severe pathological situations, particularly in the brain and liver (38). They play a safeguard role in protection against ROS, principally through having CAT, and also partly because of possessing SOD (39). Although the mitochondrial matrix is the main place of SOD, it has been reported that peroxisomal membrane enjoys its presence as well (39). However, there is no clear indication of the role of peroxisomes in neurodegenerative diseases such as HD and it is not still clear how metabolic dysfunction triggers these defects. So, it was tempting to consider its possible involvement. For this purpose, the distribution of peroxisomes was investigated by evaluating the amount of peroxisomal membrane proteins (PMP70, Pex14) along with assay of CAT activity as a peroxisomal matrix enzyme. Besides, regarding the role of peroxisome in the maintenance of redox hemostasis the activity of SOD was measured as an antioxidant enzyme. 

3-NP diminishes oxidative phosphorylation in the mitochondrial respiratory chain, reduces the level of available ATP, causing metabolic inhibition and abnormal production of ROS (40, 41). 3-NP could be used in laboratory animals to replicate the phenotype of HD associated with degeneration of the ST. Moreover, previous studies have illustrated that oxidative stress plays a fundamental role in the pathogenesis of HD (18-20). The results demonstrated that 3-NP injection reduced the activities of SOD and CAT in the ST of male rats which were in line with previous study (42). In addition to the ST, 3-NP also causes metabolic dysfunction and neuronal degeneration in other brain regions therefore, other areas including PFC, HIP, and AMY were investigated and these destructive effects of 3-NP were also observed in other studied brain areas (43). Peroxisomes are dynamic organelles, that environmental stimuli could affect dynamic regulation of their size, abundance, and function (44-46) and recently, a possible physical interaction of the Pex11 protein involved in the biogenesis of the peroxisomes with the mitochondrial Mdm34 protein has been suggested (47, 48). Mitochondrial distribution and morphology protein 34 (Mdm34)-Pex11 interaction might have dynamic functions in the regulation of ROS homeostasis, metabolite exchange between the two organelles, or the modulation of respiratory efficiency (49). Furthermore, the end product of β-oxidation in human peroxisomes, shortened fatty acyl-CoA, is shuttled into mitochondria to fuel the Krebs cycle and respiration and therefore it can be assumed that the mitochondrial respiratory chain extensively supply the ATP to peroxisomal α-oxidation pathway (3-5). As a result, the efficient function of peroxisome is necessary to avoid oxidative damage (6). Our study showed that 3-NP injection significantly reduced the level of both Pex14 and PMP70 in different brain regions. Pex14 is a membrane-anchored peroxin, involved in the peroxisomal import of matrix proteins and considers as a marker for peroxisomal number (36, 50 and 52). Recently it has been demonstrated that Pex14 has been participated in microtubule-based peroxisome motility (53). Berger’s group demonstrated a correlation between increased peroxisomal volume density and impaired peroxisome trafficking in neurons of human AD brain, associated with (54). With this in mind, our study demonstrated that 3-NP injection decreased the level of this protein and impaired peroxisomes function. This likely explains that peroxisomal trafficking dysfunction may contribute to the 3-NP-induced neurotoxicity. PMP70, as a major component of mammalian peroxisomal membranes, is now accepted as a good candidate for the overall size of peroxisomal population (55). This ATP binding cassette transporter has the duty of transporting long and branched-chain fatty acyl-CoAs so is an important factor in metabolic function of peroxisomes (37). Fanelli *et al*. indicated the specific increase in early AD of PMP70 that could reflect the need for a more efficient acyl-CoA β-oxidation (56). This increase is a compensatory mechanism in response to Aβ-induced mitochondrial dysfunction and energy metabolism (57, 58). They showed that in transgenic mice of ages between 3 and 6 months, when several hallmarks of AD pathology appeared, PMP70 is significantly decreased that indicating decreased efficiency of peroxisomal β-oxidation. In this regard, our study showed that after the development of symptoms in 3-NP-induced HD-like model, the expression level of PMP70 decreased suggesting that mitochondrial dysfunction upon 3-NP injection results in peroxisomal dysfunction and finally decreased PMP70 level. According to our results, OXT pretreatment in 3-NP-induced neural toxicity model, significantly increased the antioxidant capability through increasing the activity of the CAT and SOD enzymes. This effect appeared in different brain regions that was affected by 3-NP, which is in agreement with previous reports describing the antioxidant effects of OXT (25-27). In addition, it has been shown OXT increases glucose uptake in cardiomyocytes in critical situations such as hypoxia through calcium-calmodulin kinase (Ca-CAMKK) and AMP-activated protein kinase (AMPK) pathways (59). AMPK activation promotes glucose uptake, glycolysis, limits apoptosis, and cell damage (60, 61). Altogether, our findings suggest that OXT polished the function of PMP70 and Pex14 perhaps by improving mitochondrial function. This protective effect of OXT consequently, improves antioxidant system capability through enhancing the CAT and SOD activities that play central roles in the predominant antioxidant activity of peroxisomes and mitochondries in mammalian cells. Furthermore, it has been reported that OXT exerts its effects in a sex and context dependent manner (62, 63). Also, it has been previously described that the male rats have a commonly higher rate of hepatic β-oxidation when compared with females (64). Therefore, we asked whether the male and female rats respond differently to OXT. Our results showed no sex-dependent effect in the presence of OXT. However, many studies have addressed its sex-related effects. We do not have a clear-cut explanation for these inconsistent observations, but it could be hypothesized that the context-dependency effects of OXT is an important factor in its sex-dependent effects. Secondly, most of the measured factors in this study are metabolism-related, in a way that their abundance is strongly dependent on cellular condition and variation in lipid metabolism (3, 5). So it seems that aforementioned question could be answered better with investigating some other structural proteins in peroxisome and of course with choosing different contexts for male and female rats. This work is now in progress in our lab.
